# Do pit-building predators prefer or avoid barriers? Wormlions' preference for walls depends on light conditions

**DOI:** 10.1038/s41598-020-67979-3

**Published:** 2020-07-02

**Authors:** Inon Scharf, Akiva Silberklang, Bar Avidov, Aziz Subach

**Affiliations:** School of Zoology, Faculty of Life Sciences, Tel Aviv University, 69978 Tel Aviv, Israel

**Keywords:** Urban ecology, Behavioural ecology, Animal behaviour, Entomology

## Abstract

Ambush site selection by sit-and-wait predators is a complex process, involving biotic and abiotic considerations, which greatly affect hunting success and costs. Wormlions are fly larvae that dig pit-traps in loose soil and hunt the arthropod prey falling into their pits. They are abundant in urban environments, found below buildings that provide cover, and many of their pits are dug adjacent to walls. We examined here under what conditions wormlions prefer to dig their pits next to walls. We analysed our dataset in two ways: frequency comparisons among the different treatment combinations and a simulation null model assuming random movement. While the frequency comparisons suggested that wormlions avoided the walls under some cases, the simulation null model suggested that a combination of shallow sand and strong light in the centre led to an attraction towards the walls, independent of the wormlions’ initial location. We suggest that wall attraction results from the certain amount of shade the walls provide. We also demonstrate that shallow sand and strong illumination are unfavourable microhabitats, either leading to more frequent movement or the digging of smaller pits. We locate our results within the broader context of sit-and-wait predators and of animals’ attraction to barriers.

## Introduction

In contrast to widely-foraging predators, sit-and-wait predators do not search for prey. Rather, they choose an ambush location and wait for the prey to enter their detection range before attacking it^1,2^. This foraging mode allows sit-and-wait predators to save the energetic costs of searching and to survive long periods of shortage of prey by reducing their metabolic rate^3–5^. Ambushing prey instead of searching can also lower the risk of predation on the sit-and-wait predator, due to the positive association between searching intensity and predation risk^6–8^. This foraging mode’s drawback, however, lies in the low encounter rate with prey, forcing such predators to be opportunistic foragers^1,9^.

Sit-and-wait predators must choose their ambush sites carefully. Because sit-and-wait predators count on the prey to reach them, they often select ambush sites rich in prey in order to maximize capture success^10–12^. Sit-and-wait predators are therefore attracted to abiotic cues, which are either correlated with prey abundance or facilitate prey capture^13–16^. However, in many other sit-and-wait predators, considerations related to minimizing ambush costs dominate over those related to high prey abundance. For example, sit-and-wait predators select sites that limit exposure to extreme thermal conditions^17–20^.

Trap-building predators (hereafter, TBPs) are a sub-group of sit-and-wait predators, which construct traps to facilitate the capture of prey^21,22^. The most common trap-building predator groups are the web-building spiders and pit-building antlions or wormlions, presenting case studies of convergent evolution^21,23^. The selection of suitable sites for trap construction and ambush for prey is even more important for this group of predators than for other sit-and-wait predators, owing to the investment in trap construction. This investment is expressed in time, higher metabolic rate during construction and in self-production of building materials in spiders^22,24–27^. Due to the high investment in trap construction and the risk in movement, most TBPs do not easily relocate their trap after building it^28,29^, which makes the choice of trap location even more important. When the trap-building predators are larvae (antlions and wormlions), the ovipositing female is the one choosing the initial location of the trap. While she can choose the most suitable substrate^30^, this does not always hold true^31^. It is therefore important for the larvae to be able to later relocate, differentiate between possible options, and choose the most suitable microhabitat for trap construction.

Similar to other sit-and-wait predators, TBPs balance their selection of ambush sites between the need to capture prey and the need to avoid unfavourable abiotic conditions, such as exposure to extreme temperatures or sites that restrict the ability to construct a functional trap^32,33^. For such reasons, TBPs often prefer specific natural or artificial structures. For example, web-building spider species prefer vegetation of a specific height, thorny plants or vegetation of a specific complexity level^34–36^. TBPs are sometimes attracted to natural barriers, such as rocks, cliffs or tree trunks^37–40^. Similarly, some spiders are more frequently found next to or prefer to construct their webs on man-made walls or fences^41–44^. Such a preference for natural or artificial barriers has been explained by better thermal/humidity conditions next to the barrier, the reflection of light from a bright barrier that attracts potential prey, protection against rain, or help in the stabilization of the trap.

**Figure 1:**
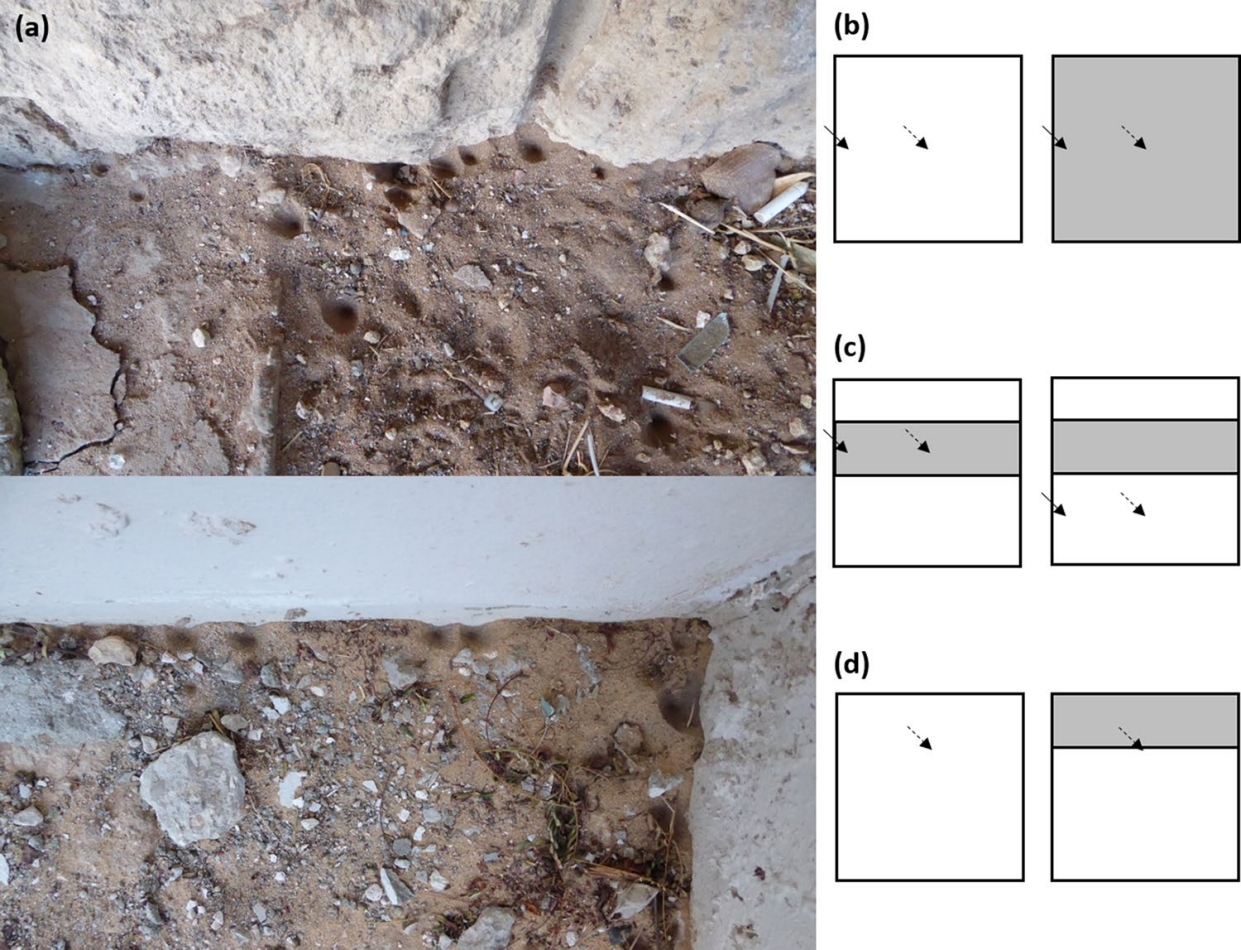
(**a**) Photos of wormlion pits in an urban habitat (Tel Aviv University, photographed by IS). Wormlion pits are often constructed adjacent to building walls. (**b**–**d**) Schemes of the experimental designs. We used arenas of 15 × 15 cm for all experiments. Wormlions were always placed individually in each arena. (**b**) Experiment 1: wormlions were placed either in shallow sand (0.5 cm depth; left) or deep sand (2 cm depth; right), either in the arena centre (dashed arrow) or next to the arena wall (continuous arrow). Experiment 3 was designed similarly to Experiment 1, but instead of a sand-depth treatment with two levels, we either exposed arenas to full light or covered them to induce full shade. (**c**) Experiment 2: The arena included a stripe of deep sand (rectangular grey area) surrounded by shallow sand. Wormlions were placed either in deep sand (left) or shallow sand (right). They were either placed next to the wall (continuous arrow) or in the arena centre (dashed arrow). Experiment 4 was designed similarly to Experiment 2, but instead of a sand-depth treatment, we used a rectangular-shaped cover providing full shade (the grey area) surrounded by full exposure to light in the rest of the arena. (**d**) The follow-up experiment comprised two treatments: the arena was fully exposed to light or one of its walls provided a 3-cm stripe of shade. Wormlions were individually placed 3 cm from the wall (arrows).

Wormlions are common pit-building predators in the Mediterranean region and are highly abundant in cities^23,45^. Wormlion pits in urban environments are often attached to or constructed close to walls (Fig. 1a). The goal of this study is to examine whether wormlions in urban habitats indeed prefer to dig their pits next to walls and under what circumstances. Since cities provide many available walls, understanding the preference of wormlions for walls can improve our understanding of why they are so successful in cities. A previous study already suggested that wormlions are abundant in cities owing to the availability of small prey and loose substrate^46^_,_ but other factors may play an important role as well. Urban habitats are the fastest-growing habitat type on earth, and while most species have higher fitness in natural habitats, some "urban specialists" are flourishing there^47,48^. Understanding therefore how such species take advantage of urban habitats is a worthy research direction^49^. While wormlions may construct pits next to walls because prey is more abundant there, we presume the reason to be an abiotic consideration and focus on the latter. This assumption is because the ambush site selection of the ecologically similar pit-building antlions is not affected by prey abundance but is strongly dictated by abiotic factors^29,50–52^. It is already known that wormlions prefer shaded, dry, and obstacle-free microhabitats of deep and fine-grained sand^53–56^.

To better understand whether and under what conditions wormlions prefer the test arena walls, we examined their preference for walls in five laboratory experiments. In the two first experiments, we explored whether unfavourable conditions, i.e., shallow sand and illumination, increase the preference for walls compared to favourable conditions – deep sand and shadow. In the next two experiments, we examined whether wormlions trade-off between proximity to walls and deep sand and between proximity to walls and shade. In other words, we sought to separate between the attraction to shade or deep sand and the possible attraction towards the test arena walls. In the fifth experiment, we examined whether shade-providing walls are preferred over walls that provide only a little shade, to support a possible link between the preference to walls and shade. We chose to test the proximity to walls in relation to shade and sand depth, because walls can at least partially/temporally provide shade, prevent excess heating and desiccation, and should therefore attract wormlions. Furthermore, sand or loose soil may be blown by the wind and accumulate next to walls (which function similarly to a "sand fence"^57^), providing a suitable substrate for wormlions' pits. However, walls may also facilitate the accumulation of leaf litter, presenting a potential disturbance for pit-building predators^58^, and possibly restrict the movement of potential prey.

We expected wormlions to prefer walls over more open areas, based on our observations in their urban habitat and the plausible advantages walls provide. This preference should become stronger under unfavourable conditions, as wormlions become more explorative in order to locate a more suitable microhabitat and because the advantages walls provide in such cases become clearer. Habitat selection decisions are not made in isolation, and wormlions need to consider the habitat preference of competitors. The first stage, nevertheless, should be the understanding the factors affecting habitat preference and how individuals respond when such factors run counter to each other.

## Materials and methods

Wormlions (Diptera: Vermileonidae) are small fly species, whose larvae dig pit-traps in loose soil and hunt the arthropod prey that falls into the pit^59,60^. In Israel, they are highly abundant in cities, found below some shelter providing cover from direct sunlight and rain^46^. They occur in clusters of high densities, higher than the ecologically-similar antlions^23^. While the studied species has not yet been described, there is only a single wormlion species in Israel. Wormlions were collected under man-made shelters at Tel Aviv University (32°6′45″ N, 34°48′15″ E) and weighed using an analytical scale (accuracy of 0.1 mg). Following all experiments (three days at the most), which were not harmful to the wormlions in any way, we released them back at their collection site. No permissions were required for this study. All laboratory experiments were conducted in a climate-controlled room (26–28 °C) and we always used fine sand (particle size < 250 µm) owing to the wormlions’ preference for such sand^53^. In Experiments 1 and 2, wormlions were kept in shade under a photoperiod of 12:12 L:D. In Experiments 3–5, they were kept in constant light with covers (or walls in Experiment 5) being used to provide constant shade according to the specific treatment. We used identical arenas of 15 × 15 cm in all experiments. There was no difference in wormlion body mass among any treatments within each experiment (P > 0.64). The movement distance of each wormlion was estimated by dividing the test arena into 100 equal squares and counting the number of squares traversed by the wormlions (similar to^61^). We used this method rather than following wormlion movement, because when wormlions move over long distances, newer tracks cross the older ones, hindering an exact estimation of movement distance. The wormlion pit area was measured from photos using ImageJ^62^. Experiments were conducted between December 2019 and March 2020.

### Experiment 1: the effect of sand depth on the preference for the test arena walls

We collected 100 wormlions, weighed and allocated them individually to one of two sand-depth treatments: deep sand (2 cm sand) or shallow sand (0.5 cm sand). The wormlions were placed either in the arena centre or next to one of the arena’s four walls, in a full-factorial design (two sand-depth treatments × two initial locations; Fig. 1b). After 24 h, we photographed each arena and documented whether the wormlions had moved or whether they had retained their initial location relative to the wall (e.g., remained next to the wall when placed there) or changed it (moved from the arena centre towards the wall or vice versa). We also estimated the movement distance and measured pit area, as described above.

### Experiment 2: separation between the preference for deep sand and the test arena walls

We collected 100 wormlions and placed them individually in a test arena. The arena comprised mostly of shallow sand, except for a stripe of a 3-cm width, providing deep sand (Fig. 1c). We had four treatment combinations according to the initial location: placing the wormlion on either deep or shallow sand, and either next to the arena wall or in the arena centre. On shallow sand, wormlions were placed 2 cm away from deep sand. After 24 h, we photographed each arena and measured the same variables as in Experiment 1. The purpose in this full-factorial design was to separate between a possible preference for deep sand from a preference for walls and the difference between this experiment and the previous one lies in that here wormlions could choose between sand depths. In addition to comparing the similarity between the final and initial location relative to the wall (remained or changed), we also compared the final and initial location relative to sand depth (remained or changed).

### Experiment 3: the effect of illumination on the preference for the test arena walls

We collected 100 wormlions and allocated them individually to one of two illumination treatments (constant light or constant shade; Fig. 1b). We used only shallow sand because the previous experiment had suggested that movement, and hence habitat choice, is greater in shallow sand (see “Results”). The wormlions were placed either in the arena centre or next to one of its walls, in a full-factorial design. After 24 h, we photographed each arena and measured the same variables as in Experiment 1.

### Experiment 4: separation between the preference for shade and the test arena walls

We collected 100 wormlions and placed them individually in a test arena with a 3-cm width cover, placed 2 cm above the sand, providing shade (Fig. 1c). We used shallow sand. We had four treatment combinations according to the initial location: placing the wormlion either under shade or exposed to light, and either next to the arena wall or in the arena centre. Under light, wormlions were placed 2 cm away from shade. After 24 h, we photographed each arena and measured the same variables as in Experiment 1. In addition to comparing the similarity between the final and initial location relative to the wall (remained or changed), we also compared the final and initial location relative to the shade (remained or changed). The difference between this experiment and the previous one lies in that here wormlions could change their proximity to the wall and move from light to shade or vice versa.

### Simulation model: null hypothesis for the preference for the test arena walls

If we assume that wormlions display random walk (an equal probability to move in any possible direction), the probability of wormlions placed in the arena centre reaching the wall when moving is lower than the probability of wormlions placed next to the wall to move away from it. The reason for this is that the arena periphery occupies a smaller area than its centre. Furthermore, a wormlion placed in the centre has better access to central squares compared to a wormlion placed at the edge, which has poor access to most of the edge squares. Finally, simple statistics ignores the differences in movement distance among treatments. Simple statistics may therefore fail by indicating differences between treatments in the final location, whereas these differences can be explained based only on the initial location and movement distance. In order to provide a more realistic prediction, or a ‘null model’, for the similarity between the initial and final locations relative to the wall, we designed a simulation model in MATLAB (R2017) (see Fig. S1 in the Supplementary Material for the simulation’s chart flow). We used the initial location (arena centre or next to the wall) and moved each wormlion for the observed movement distance in the relevant treatment combination. We referred to the movement as the number of squares covered of the 100 squares in the arena, in order to compare the simulation results with the experimental data. In addition, a square of 2.25 cm^2^ enables the construction of a circular pit of 1.77 cm^2^. This value is slightly smaller than the median area of pits constructed in shallow sand based on Experiments 3 and 4 (1.92 cm^2^). Pits constructed in deep sand are typically larger. Therefore, the vast majority of pits constructed in any peripheral square are attached to the wall. Movement direction was decided randomly and independently for each simulation step (8 possible directions: down, up, right and left, and any combination of the former two with the latter two), until the movement distance was reached (i.e., the number of squares visited). We then noted whether the simulated wormlion was present in the peripheral squares (the arena envelope, 36 squares) or its centre (64 squares). If the initial and final locations were identical, we recorded a value of “1”, and if not, a value of “0”. The proportion of similar locations (ones) out of the sample size for each treatment combination was used as the expected value. We ran each simulation 1,000 times and calculated the mean and the 95% confidence intervals for each treatment combination. If the observed proportion, including its standard error, fell within the confidence intervals, we determined that the final location did not depend on the existence of a wall, but only on movement distance and random movement direction. If not, it indicated that wormlions were located next to the wall or in the arena centre more than randomly expected. Our simulation model is similar to a bootstrap procedure, meant to determine the confidence intervals for an unknown distribution^63^.

### A follow-up experiment: attraction to shade-providing walls

Our results indicate that wormlions under light conditions prefer to construct pits next to the walls more frequently than those under shade (see “Results”). We suggest that even under full light conditions, the attraction to the wall is caused by the partial shade they still provide (see “Discussion”). We therefore designed a small follow-up experiment to examine whether shade-providing walls are more attractive to wormlions than those that do not provide shade. We collected 78 wormlions and placed them individually in a test arena. We used table lamps as a light source in an angle of 45° (between the ground and the bulb), 30 cm above the arena. We applied two treatments: lit arenas and arenas in which a 3-cm stripe adjacent to the wall was shaded. To simulate shading by walls, we used vertical 8-cm height plastic “walls”, attached to the arenas (Fig. 1d; see Supplementary Material, Fig. S2, for a photo). We used here 2-cm deep sand. Wormlions in both treatments were placed 3 cm from the wall, in the arena’s centre. In the treatment with the shade-providing wall, the initial location was at the border between the lit and shaded parts of the arena. After 24 h, we photographed each arena and documented whether the pit constructed was adjacent to the wall, they were placed next to.

### Statistical analysis

The two categorical response variables, movement (y/n) and final location relative to the initial one (remained/changed) were analysed using χ^2^ tests with the four treatment combinations. When the result of this analysis was significant, we used six separate χ^2^ tests between each pair of treatment combinations, controlling for the false discovery rate^64^, to detect which treatment combinations differed from each other. The two continuous variables, pit area and movement distance, were analysed using an ANCOVA, with each experiment's two treatments as the explanatory variables and body mass as a covariate. Regarding the pit area, we considered it more informative to examine the effect of the final/chosen location instead of the initial one (the final location should affect the pit area more). When the two-way interaction of the two explanatory variables was not significant it was removed and the test was redone. Pit area and movement distance were square-root- and log_10_-transformed, respectively, due to their deviation from a normal distribution. Statistical analyses were conducted in SYSTAT v. 13.

## Results

### Experiment 1: the effect of sand depth on the preference for the test arena walls

**Figure 2:**
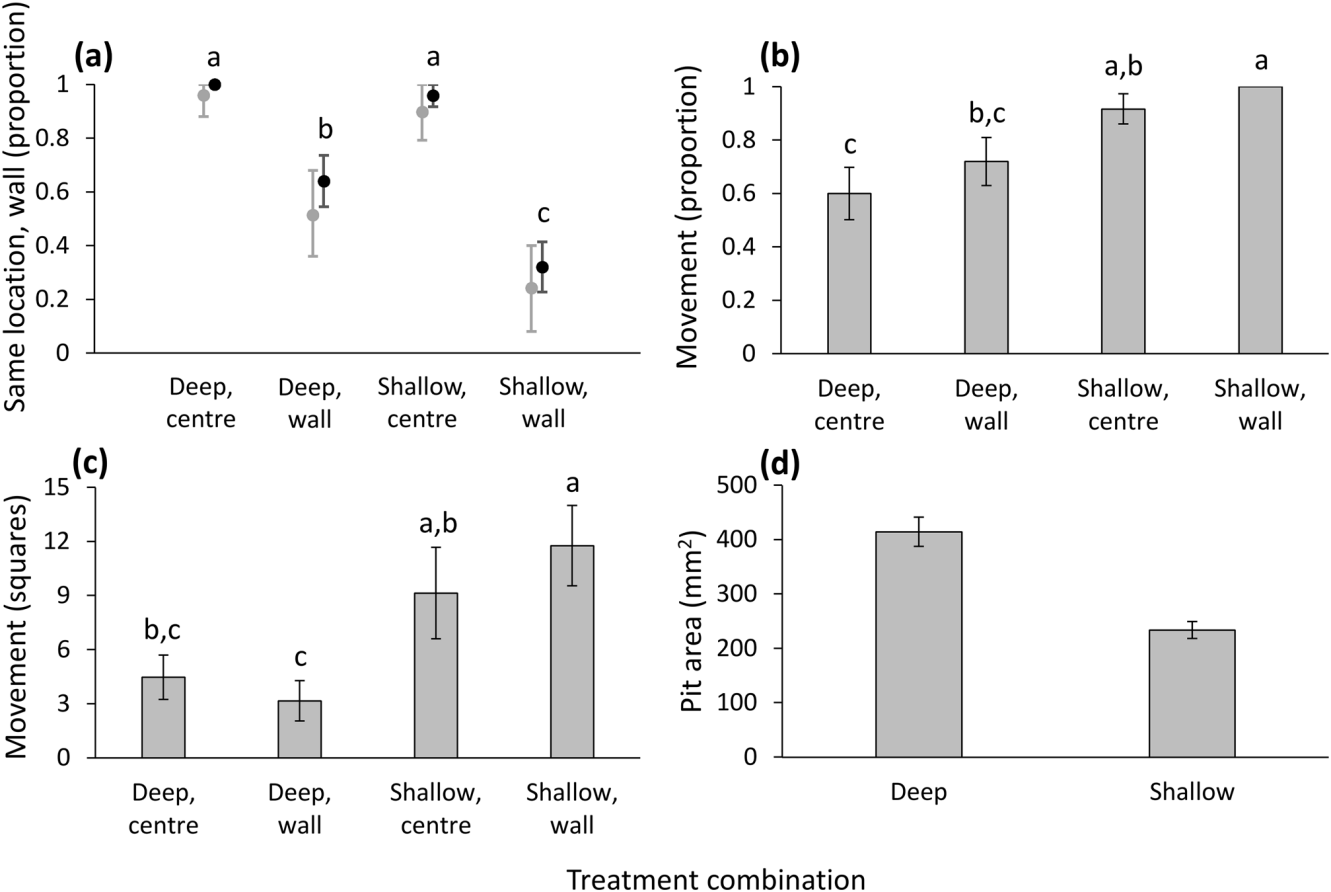
Results of Experiment 1: The effect of sand depth on the preference for the test arena walls. (**a**) Black circles and error bars: The proportion of wormlions remaining in their initial location relative to the wall according to the four treatment combinations. Grey circles and error bars: The expected similarities between the initial and final location relative to the wall, based on the simulation null model. (**b**) The proportion of wormlions that moved during the experiment according to the four treatment combinations. (**c**) The movement distance (measured as the number of squares out of 100) of wormlions. (**d**) Pit area in deep vs. shallow sand. Error bars stand for 1 SE, calculated for proportions according to the formula: $\sqrt{\frac{p(1-p)}{n}}$. Letters stand for differences among treatments based on post-hoc comparisons. The grey error bars in (**a**) stand for the 95% confidence intervals.

Whether the wormlion remained in its initial location relative to the wall or changed it depended on its original location (χ^2^ = 37.702, df = 3, n = 99, P < 0.001; Fig. 2a). Wormlions placed in the arena centre almost always remained there, irrespective of whether the sand was deep or shallow, while those placed next to the wall moved away more frequently. Initial location next to the wall in shallow sand induced more change in the final location (from next to the wall to the arena centre) than the initial location in deep sand. In contrast, the simulation model predicted well the similarity in final location relative to the wall, given the initial location and the movement distances in each treatment (Fig. 2a). The tendency to move was mostly affected by sand depth (a lower tendency to move in deep sand) but also by the initial location (a higher tendency to move when placed next to the wall) (χ^2^ = 15.992, df = 3, P = 0.001; Fig. 2b). In regard to movement distance, there was an interaction between sand depth and initial location (F_1,75_ = 4.333, P = 0.041; Fig. 2c): wormlions placed next to the wall in shallow sand moved over slightly longer distances than those placed in the arena centre, while the opposite held true in deep sand. Generally, wormlions moved over longer distances in shallow sand (F_1,75_ = 16.179, P < 0.001), while location next to the wall had no effect as a main effect (F_1,75_ = 0.083, P = 0.774). Body mass had a positive effect on movement (F_1,75_ = 8.013, P = 0.006). Pit area was positively affected by body mass (F_1,93_ = 30.483, P < 0.001), and was larger in deep sand than shallow sand (F_1,93_ = 38.767, P < 0.001; Fig. 2d). Final location regarding the wall had no effect on pit dimensions (F_1,93_ = 1.241, P = 0.268). The two-way interaction was not significant and hence removed (F_1,92_ = 0.934, P = 0.336).

### Experiment 2: separation between the preference for deep sand and the test arena walls

**Figure 3:**
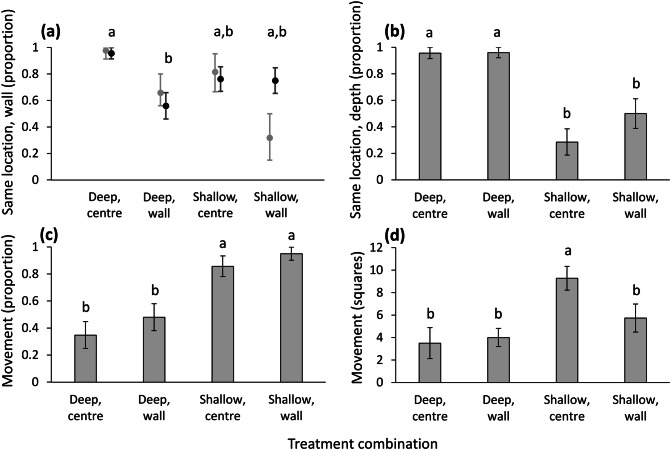
Results of Experiment 2: Separation between the preference for deep sand and the test arena walls. (**a**) Black circles and error bars: The proportion of wormlions remaining in their initial location relative to the wall according to the four treatment combinations. Grey circles and error bars: The expected similarities between the initial and final location relative to the wall, based on the simulation null model. (**b**) The proportion of wormlions remaining in their initial location relative to the four treatment combinations. (**c**) The proportion of wormlions that moved during the experiment. (**d**) The movement distance (number of squares) of wormlions according to the four treatment combinations. Error bars stand for 1 SE. Letters stand for differences among treatments based on post-hoc comparisons. The grey error bars in (**a**) stand for the 95% confidence intervals.

The similarity between final and initial location relative to the wall differed among treatments (χ^2^ = 10.134, df = 3, n = 89, P = 0.017; Fig. 3a): wormlions placed in deep sand next to the wall changed their location the most, while those placed in deep sand in the centre changed their location the least. The simulation indicated that the deviant treatment was rather shallow sand, next to the wall: only when wormlions were placed there, they were closer to the wall than expected after the experiment (Fig. 3a). The final location considering sand depth differed greatly among treatments: while wormlions initially placed in deep sand remained there, those placed in shallow sand mostly moved to deep sand (χ^2^ = 35.993, df = 3, P < 0.001; Fig. 3b). There was no difference between wormlions ending up in deep and shallow sand in their final location next to the wall (χ^2^ = 1.077, df = 1, P = 0.299). The tendency to move differed among treatments (χ^2^ = 23.952, df = 3, P < 0.001; Fig. 3c): wormlions placed in shallow sand moved more than those placed in deep sand, irrespective of their location relative to the wall. The initial location regarding sand depth and the wall interacted to affect movement distances (F_1,52_ = 5.177, P = 0.027; Fig. 3d): movement distances of wormlions placed in shallow sand in the centre were greater than all other treatments. Movement was positively affected by body mass (F_1,52_ = 6.718, P = 0.012). To complete the statistical report, initial location regarding the wall was not significant as main effect, but sand depth was (F_1,52_ = 1.634, P = 0.207 and F_1,52_ = 10.218, P = 0.002, respectively). Pit area was positively affected by the sand depth chosen (larger in deep sand: F_1,79_ = 66.525, P < 0.001), body mass (F_1,79_ = 9.354, P = 0.003), but not by the final location regarding the wall (F_1,79_ = 2.549, P = 0.114), or the two-way interaction (F_1,78_ = 0.385, P = 0.537).

### Experiment 3: the effect of illumination on the preference for the test arena walls

**Figure 4:**
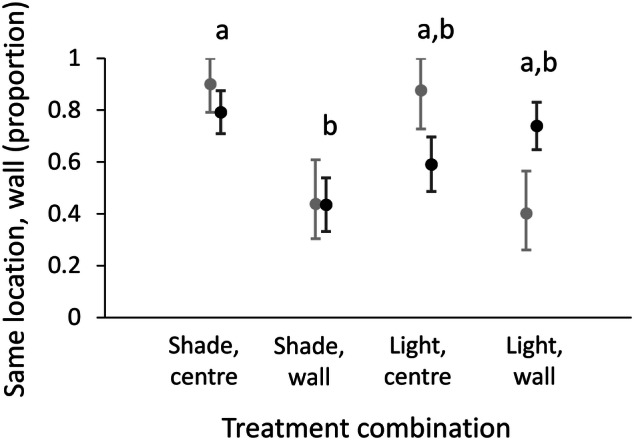
Results of Experiment 3: The effect of illumination on the preference for the test arena walls. Black circles and error bars: The proportion (± 1 SE) of wormlions remaining in their initial location relative to the wall according to the four treatment combinations. Grey circles and error bars: The expected similarities between the initial and final location relative to the wall, based on the simulation null model (and 95% confidence intervals). Letters stand for differences among treatments based on post-hoc comparisons. The post-hoc comparison is not significant after the correction for the false discovery rate, but the marked difference is the largest one and the analysis of all treatment combinations is significant.

The similarity between the initial and final location relative to the shade differed among treatments (χ^2^ = 7.823, df = 3, n = 92, P = 0.050; Fig. 4). Pairwise comparisons indicated that the difference was under shade between initial location next to the wall and in the arena centre: while those placed in the centre mostly remained there, the majority of those placed next to the wall moved away from it. In contrast, the simulation indicated that under light, wormlions ended closer to the wall than expected, irrespective of their initial location (Fig. 4). There was no difference among the four treatment combinations in the tendency to move (χ^2^ = 3.083, df = 3, P = 0.379). Regarding movement distances, neither body mass nor initial location nor illumination affected movement distance (mass: F_1,67_ = 1.144, P = 0.289; initial location: F_1,67_ = 0.527, P = 0.470; illumination: F_1,67_ = 0.941, P = 0.336; two-way interaction: F_1,66_ = 0.760, P = 0.386). Pit area was positively correlated only with body mass (F_1,71_ = 28.804, P < 0.001), with no effect of illumination (F_1,71_ = 1.043, P = 0.311), final location regarding the wall (F_1,71_ = 1.698, P = 0.197), or the two-way interaction (F_1,70_ = 2.106, P = 0.151).

### Experiment 4: separation between the preference for shade and the test arena walls

**Figure 5:**
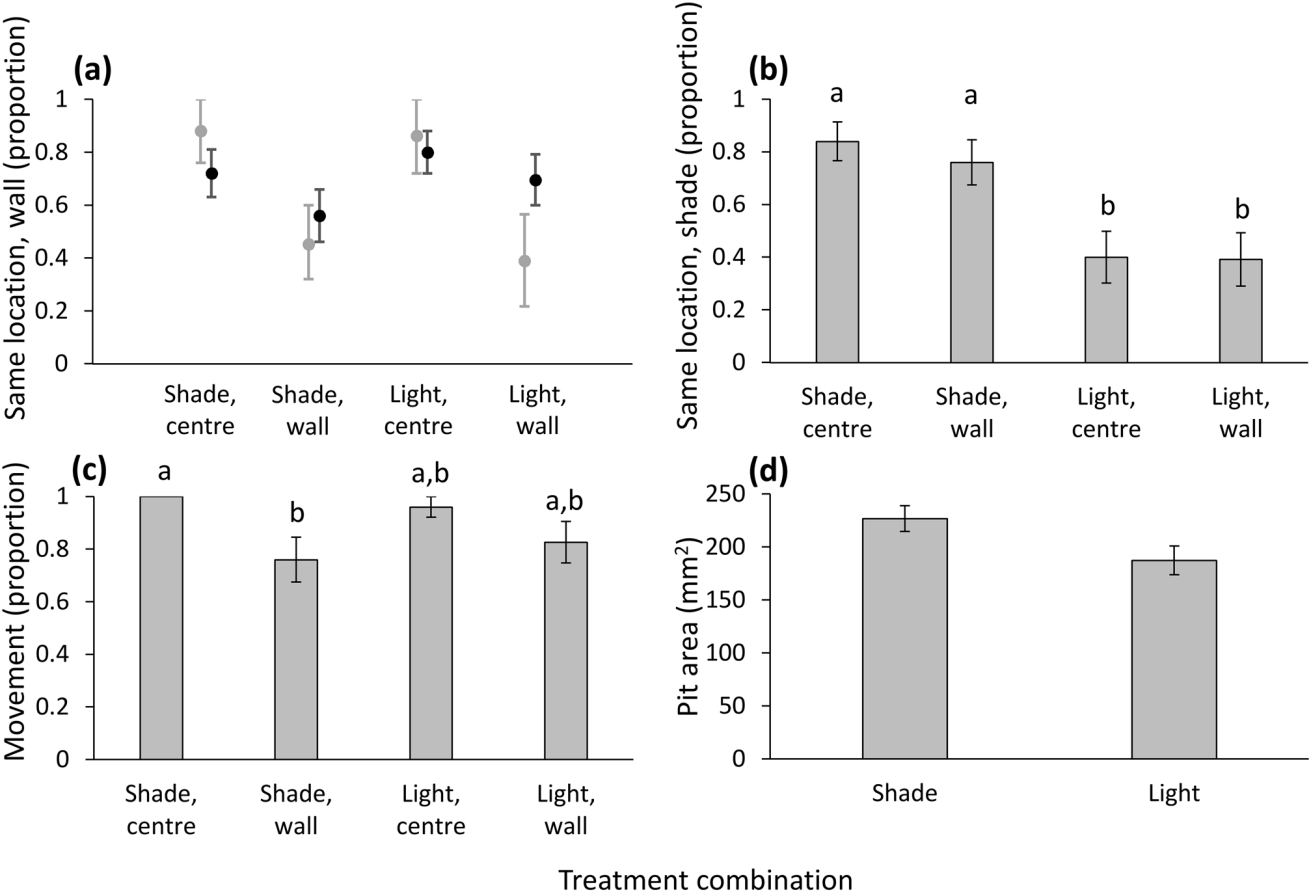
Results of Experiment 4: Separation between the preference for shade and the test arena walls. (**a**) Black circles and error bars: The proportion of wormlions remaining in their initial location relative to the wall according to the four treatment combinations. Grey circles and error bars: The expected similarities between the initial and final location relative to the wall, based on the simulation null model. (**b**) The proportion of wormlions remaining in their initial location relative to shade. (**c**) The proportion of wormlions that moved during the experiment. (**d**) The Pit area under shade vs. light. Error bars stand for 1 SE. Letters stand for differences among treatments based on post-hoc comparisons. The grey error bars in (**a**) stand for the 95% confidence intervals. The post-hoc comparison in (**c**) is not significant after the correction for the false discovery rate, but the marked difference is the largest one and the analysis of all treatment combinations is significant.

The four treatment combinations did not differ in the similarity between the initial and final location relative to the wall (χ^2^ = 3.516, df = 3, n = 98, P = 0.319). However, wormlions placed next to the wall under light remained there more than expected, according to the simulation (Fig. 5a). The similarity between the initial and final location relative to the shade differed among the four treatment combinations (χ^2^ = 17.035, df = 3, P < 0.001; Fig. 5b). The difference was between the initial location relative to shade: while those initially placed under shade usually remained there, those placed under light usually switched to shade. Wormlions ending up under light were more frequently next to the wall than those ending up under shade (72% vs. 30% next to the wall under light and shade, respectively; χ^2^ = 14.693, df = 1, P < 0.001). The tendency to move differed among the four treatment combinations (χ^2^ = 9.443, df = 3, P = 0.024; Fig. 5c). Under shade conditions, those placed next to the wall moved less frequently than those in the centre. Movement distance was positively correlated with mass (F_1,83_ = 32.948, P < 0.001), but with neither of the two other treatments (initial location regarding illumination: F_1,83_ = 0.031, P = 0.861; initial location regarding the wall: F_1,83_ = 1.361, P = 0.247; two-way interaction: F_1,82_ = 0.807, P = 0.372). Regarding pit area, pits constructed under shade were larger (F_1,85_ = 5.670, P = 0.019; Fig. 5d), and larger wormlions constructed larger pits (F_1,85_ = 6.224, P = 0.015). Neither the final location regarding the wall nor the two-way interaction influenced pit area (F_1,85_ = 0.528, P = 0.470 and F_1,84_ = 0.116, P = 0.734, respectively).

### A follow-up experiment: attraction to shade-providing walls

Wormlions ended up more frequently next to the wall when the wall was providing shade than in the treatment in which walls did not provide shade (χ^2^ = 6.271, df = 1, n = 78, P = 0.012; 59% vs. 31%, respectively).

## Discussion

The selection of a suitable ambush site for trap-building predators (TBPs) is of great importance, owing to its consequences for growth and survival and due to the high cost of relocating and abandoning an existing trap. We examined here whether wormlions as TBPs preferred to dig a pit-trap next to the test arena walls or in the centre. The walls represented barriers in the wormlions’ habitat. We tested such possible preference under either favourable conditions (deep sand, shade) vs. unfavourable conditions (shallow sand, light) and combined these conditions with the initial location (next to the wall or the centre). We expected the walls to be preferred, especially under unfavourable conditions. The preference shown for the arena walls depended on the method of analysis, initial location, and was treatment-specific, as detailed below. The simple statistical analysis suggested a general avoidance of the wall. In contrast, the simulation null model suggested that the wormlions’ initial location and their movement distance, assuming random movement, could mostly explain their final location, with a notable exception: Wormlions were located closer to the wall at the end than expected under light conditions. Both methods of analysis contribute to our understanding of a preference for the arena walls, but while the simple analysis considers only the initial and final locations, the simulation model considers also the movement in each treatment combination and corrects for the lower area occupied by the periphery in the arena.

Simple statistical comparisons of the wormlions’ initial and final locations (next to the wall vs. not next to the wall) mostly revealed avoidance of the walls. In Experiments 1 and 3, fewer wormlions retained their initial location next to the wall than those placed in the arena centre, in both sand depths and under shade (Figs. 2a, 4). In contrast, the simulation null model indicated that most similarities between initial and final locations could have been randomly obtained, given the distance that each wormlion moved. In other words, there was usually neither attraction to nor avoidance of the wall in this experiment. An important exception to this pattern was the preference for the wall under light conditions: more wormlions remained next to the wall and more wormlions switched from the arena centre to the wall than expected by chance (Figs. 4, 5a).

Katz et al.^65^ have already detected a possible preference for the arena walls under light and suggested that the arena walls provide a certain amount of shade. After the simulation model supported this idea, we examined whether in Experiment 4, wormlions ending up under light are more frequently next to the wall than those ending up under shade. This indeed held true, supporting our explanation (note that the same analysis considering the sand depth and wall preference resulted in no significant result). Our follow-up experiment is another piece of supporting evidence because it demonstrates the increase in the preference for walls when the shade they provide is enlarged. We believe that other suggestions for wall preference, such as wall-following behaviour being a way by which to explore the habitat by vision-limited animals^66,67^, are less likely here because the wall is preferred mostly under light. Other studies suggest that wall-following behaviour is an artefact of using a too-small arena in the laboratory when animals reach fast the test arena edges and then move around them^68,69^. This explanation, which fits better more active animals than wormlions, is also unlikely because the movement distance of wormlions was quite limited (Figs. 1c, 2d). That said, we cannot completely rule out the possibility that movement over longer distances, especially in unfavourable habitats, leads to some preference for the walls, owing to wall-following movement. In any case, this could be only an additive process to the effect of light we demonstrated here because wormlions did not move over longer distances under light than shade conditions and still ended up next to the wall more frequently than expected. It is important yet to note that we have no clear evidence for wall-following movement.

Almost nothing is known about the potential predators of wormlions and it is unclear whether walls can protect against all predators or at least against some of them. As a parallel example, *Argiope* spiders prefer to construct their webs in dense vegetation rather than in more open habitats, to improve protection against bird predators^70^. We have also little knowledge of the prey composition of wormlions and whether it is more abundant next to walls. Indeed, some ant species follow walls while searching^71,72^. Wormlions prey on smaller ants than antlions^73^ and if smaller ants are more abundant next to walls than larger ants, this could be another reason attracting wormlions to the walls. That said, the ecologically similar antlions consider abiotic factors much more greatly than prey abundance when choosing a pit-trap site^29^, so it remains to be tested whether prey abundance is the reason for the wall preference under light conditions we detected.

In the most thorough study to date examining this question, two pit-building antlions were found to prefer the microhabitat’s centre, while a non-pit-builder used barriers to improve its capture success^74^. In contrast, another study suggested that pit-building antlions generally inhabit spaces closer to the edge of buildings, which perhaps provide them with a better microclimate^56^. Here, we experimentally examined the same phenomenon in wormlions and, in contrast to the observations reported in the two above-cited studies, reached a more complex conclusion regarding wall preference. Miler et al.^56^ suggest that their studied antlion species prefers the habitat edge because the sand there is drier or warmer. In our study, wormlions probably prefer to dig pits next to the walls only when the latter provide shade, to avoid too high a temperature. Wormlions prefer dry to wet microhabitats^55^. Therefore, if walls can protect against rain, this should be an additional reason for wormlions to prefer them. Our experiment had demonstrated that only light is enough for wormlions to move next to walls. This tendency may be stronger under natural conditions, owing to the thermal differences of between sand under shade and sand exposed to direct sunlight.

Wormlions moved more and over longer distances in shallow sand, constructed smaller pits in it, and moved to deep sand when a choice was given. This finding strengthens our previous suggestion that shallow sand is considered unfavourable for pit-building predators^61^, either because smaller pits must be constructed or due to slower response to prey there. Direct light is also unfavourable, and wormlions avoided it in our experiment and constructed smaller pits there. The switch from light to shade when placed 2 cm away from shade took place in most of the cases, irrespective of location relative to the wall. In this experiment, there was hierarchical decision-making: shade was preferred over light and when shade could not be reached, there was a preference for the arena walls. Regarding nest selection, animals can either rank the nest attributes they hierarchically prefer or reach a “weighted sum of nest-site attractiveness”^75^. One example of a hierarchy, as found here, is that of the new nest attributes preferred by bees: some odours are preferred, but only in the absence of other odours higher in the hierarchy^76^.

The movement was most obviously affected by sand depth, being more frequent and over longer distances in shallow sand. Movement in other TBPs reflects disturbance or the habitat's unsuitability for the predator^77,78^. The effect of initial location (wall/centre) on movement was minor and inconsistent. For example, while wormlions moved over shorter distances when initially located next to the wall than in the centre under shade in Experiment 4, there was no such difference in Experiment 3. We conclude that movement is not strongly dictated by the initial location relative to the wall but more strongly by other factors, such as sand depth.

The disagreement between the simple statistical analysis and the null model emphasizes the importance of null models in analysing animal behaviour and spatial data. For instance, ecologists should not assume a uniform resource-use pattern of the habitat of central-place foragers but should instead assume a higher probability of their using the areas next to their central place than more distant areas^79^. More generally, when measuring preference for certain habitats, it is necessary to account for their relative accessibility to the studied species^80^. Our simulation model differs from the simple statistical analysis by two important features: The simulation considers the distinct movement distance of each treatment combination and the fact that the arena's periphery is smaller in its area than the arena's centre. Like all models, our simulation model has some simplifying assumptions, such as the equal probability of moving in all directions and the treatment of the arena as being fully homogenous. Both are probably wrong, but we aimed at providing the simplest null model possible.

After we determined the preference for walls when they provide shade, future research should examine whether walls can provide long-term fitness benefits for wormlions in urban habitats. For example, it will be interesting to examine whether walls improve the wormlion survival under high temperatures and whether more prey move next to walls, providing better predation opportunities for wormlions. While we used constant light in the laboratory, results in the field may differ, as walls can provide shade only during some part of the day due to the sun's movement. Therefore, south-facing walls (in the northern hemisphere) should be more effective in shading than north-facing ones. While here we focused on the preference of individual wormlions, it will be interesting to study how microhabitat preferences change with density, and what the identity of the individuals that keep the preferred microhabitats is.

## Supplementary information

Supplementary file1
